# Influence of the Addition of Zinc, Strontium, or Magnesium Oxides to the Bioglass 45S5 Network on Electrical Behavior

**DOI:** 10.3390/ma17020499

**Published:** 2024-01-20

**Authors:** Sílvia Rodrigues Gavinho, Imen Hammami, Suresh Kumar Jakka, Sílvia Soreto Teixeira, Jorge Carvalho Silva, João Paulo Borges, Manuel Pedro Fernandes Graça

**Affiliations:** 1 I3N and Physics Department, Aveiro University, 3810-193 Aveiro, Portugal; silviagavinho@ua.pt (S.R.G.); imenhammami@ua.pt (I.H.); suresh@ua.pt (S.K.J.); silvia.soreto@ua.pt (S.S.T.); 2 I3N-CENIMAT and Physics Department, NOVA School of Science and Technology, Campus de Caparica, 2829-516 Caparica, Portugal; jcs@fct.unl.pt; 3 I3N-CENIMAT and Materials Science Department, NOVA School of Science and Technology, Campus de Caparica, 2829-516 Caparica, Portugal; jpb@fct.unl.pt

**Keywords:** Bioglass^®^, biomaterial, zinc, magnesium, strontium, osseointegration, electrical properties, implant coatings

## Abstract

45S5 Bioglass has been widely used in regenerative medicine due to its ability to dissolve when inserted into the body. Its typically amorphous structure allows for an ideal dissolution rate for the formation of the hydroxyapatite layer, which is important for the development of new bone. This bioactive capacity can also be controlled by adding other oxides (e.g., SrO, ZnO, and MgO) to the 45S5 Bioglass network or by storing electrical charge. Ions such as zinc, magnesium, and strontium allow for specific biological responses to be added, such as antibacterial action and the ability to increase the rate of osteoblast proliferation. The charge storage capacity allows for a higher rate of bioactivity to be achieved, allowing for faster attachment to the host bone, decreasing the patient’s recovery time. Therefore, it is necessary to understand the variation in the structure of the bioglass with regard to the amount of non-bridging oxygens (NBOs), which is important for the bioactivity rate not to be compromised, and also its influence on the electrical behavior relevant to its potential as electrical charge storage. Thus, several bioactive glass compositions were synthesized based on the 45S5 Bioglass formulation with the addition of various concentrations (0.25, 0.5, 1, and 2, mol%) of zinc, strontium, or magnesium oxides. The influence of the insertion of these oxides on the network was evaluated by studying the amount of NBOs using Raman spectroscopy and their implication on the electrical behavior. Electrical characterization was performed in ac (alternating current) and dc (direct current) regimes.

## 1. Introduction

Implants play an important role in the treatment of various medical conditions, from replacing damaged or diseased tissues to restoring lost function. While titanium has long been the material of choice due to its exceptional biocompatibility and mechanical properties, it is not without its limitations. Weak bone regeneration and the risk of instability during the healing phase have been recognized as significant challenges in the field of implantology [1,2,3]. Surface treatments that improve surface roughness have been an important strategy for increasing osseointegration, allowing for a stronger bond between the implant and the surrounding bone tissue [4,5,6]. However, this strategy may not be sufficient to assure long-term implant success, especially in situations where inflammatory reactions can result in bone support loss and implant failure [7,8].

One potential solution to these problems is to coat implants with bioactive materials [9,10,11,12]. Bioglass, composed of 46.1SiO_2_-24.4Na_2_O-26.9CaO-2.6P_2_O_5_ in mol%, was first introduced by Larry L. Hench in the 1960s and has since opened the way for numerous breakthroughs in the implant industry [13,14,15,16]. Its remarkable bioactivity, particularly in the context of coating metal implants, exceeds that of conventional biomaterials. The crucial factor to its effectiveness lies in the formation of a strong, carbonated hydroxyapatite layer that mimics the structure of the host bone, ensuring an effective chemical bond and thereby emphasizing the stability of the implant [13,17,18,19,20]. Moreover, bioglass exhibits inherent osteoinductive and osteogenic properties, stimulating the recruitment and differentiation of human primary osteoblasts, thereby enhancing their proliferation [16,21,22].

Several studies have shown that the incorporation of therapeutic agents into bioglass enhances its biological properties [23,24,25,26,27,28,29]. Zinc (Zn) is an essential nutrient element that is essential in DNA replication and plays a relevant role in cell growth, development, and differentiation [30,31]. Moreover, it has been shown to have antimicrobial activities against various microorganisms [32,33,34]. Magnesium (Mg) is an important element for cellular activity, promoting the proliferation and differentiation of osteoblasts [35,36,37,38,39]. Furthermore, its deficiency can lead to impaired bone growth, heightened trabecular bone loss, and increased bone resorption. Strontium (Sr) plays an important role in the process of bone regeneration [40,41]. Its influence extends to enhancing bone microarchitecture and elevating bone mineral density [40]. Strontium exerts biological effects by promoting osteogenesis, thereby improving the proliferation and differentiation of pre-osteoblastic cells into osteoblasts. Additionally, it hinders the process of bone resorption and osteoclast differentiation and activity, thereby avoiding osteoclastogenesis [42,43,44].

One of the key features that make bioglass an exceptional biomaterial is its ability to store large quantities of electrical charges with low polarization [45,46,47,48]. This remarkable property arises from the high mobility of sodium ion chains within its glass structure. The unique composition of bioglass allows it to accumulate and release electrical charges efficiently, which can be harnessed for various therapeutic purposes. The biological properties can be influenced by the charge type (whether it is positive or negative) and the quantity of accumulated charge [46]. It has been reported that surfaces with a negative charge promote tissue regeneration and enhance bone growth and osseointegration by stimulating osteoblast activity [49,50,51]. Yamashita et al. [45,52] explored apatite growth on crystalline hydroxyapatite (HA) and bioactive glass surfaces after electrical polarization. The surface charges influenced bioactivity, leading to enhanced calcium phosphate layer growth, increased cell proliferation, and improved osteoconductivity in vivo. Obata et al. [47,48] demonstrated that the electrical polarization of 45S5 Bioglass, influenced by sodium ion migration, could be controlled by polarization time, temperature, and applied voltage. Compared to HA, 45S5 bioglass achieved 1000 times higher polarization with 100 times lower DC field. Polarizing bioglass at 500 °C with 10 V for 1 h led to persistent surface charges, significantly enhancing bioactivity. Morphological differences in apatite layers were observed among non-polarized, positive, and negative surfaces after 2 h in simulated body fluid, but no distinctions were detected after 24 h. Verma et al. [53] established in their study that the presence of electrical charge on the 1393 bioglass surface (53 wt% SiO_2_, 20 wt% CaO, 12 wt% K_2_O, 6 wt% Na_2_O, 5 wt% MgO, 4 wt% P_2_O_5_) can affect its interaction with cells, potentially enhancing its antibacterial properties through electrostatic interaction as bacterial cells possess electric charge, and improving the proliferation and adhesion of osteoblast cells. 

This paper aims to explore the electrical properties of 45S5 bioglass and assess whether the introduction of zinc, magnesium, and strontium oxides has any adverse effects on its charge storage capabilities. For this purpose, bioglasses modified with the insertion of various concentrations of ZnO, MgO, and SrO (0.25, 0.5, 1, and 2, mol%) were synthesized using the melt-quenching technique. Using impedance spectroscopy (IS), changes in the electrical properties of the prepared glasses were verified. Furthermore, morphological characterization was performed using scanning electron microscopy (SEM), and structural characterization was assessed using X-ray diffraction (XRD) and Raman spectroscopy.

## 2. Materials and Methods

### 2.1. Bioglasses Synthesis

The bioglass base composition used in this study was prepared based on the Bioglass^®^ developed by Hench et al. (46.1SiO_2_-24.4Na_2_O-26.9CaO-2.6P_2_O_5_, mol%). The bioglass composition was modified by the incorporation of various concentrations (0.25, 0.5, 1, and 2, mol%) of ZnO (Zn0.25, Zn0.5, Zn1, and Zn2), MgO (Mg0.25, Mg0.5, Mg1, and Mg2), and SrO (Sr0.25, Sr0.5, Sr1, and Sr2). The chemical precursors, including SiO_2_, P_2_O_5_, CaCO_3_, Na_2_CO_3_, and MgO, or Sr(NO_3_)_2_ or ZnO, supplied by Sigma-Aldrich, Darmstadt, Germany, with a high purity grade (≥99%), were mixed and homogenized using a planetary ball-milling process for 1 h at 300 rpm. The obtained powders were calcined for 8 h at 800 °C and then melted in a platinum crucible at 1300 °C for 1 h. To ensure greater sample homogeneity, the bioactive glass was re-melted using the same parameters. To control the thickness of the samples, the molten glass was quenched between two casting plates, at room temperature, to obtain bulk glass samples. After, the obtained samples were polished to achieve uniform dimensions of approximately 1 mm in thickness.

### 2.2. Structural and Morphological Characterizations

Structural characterization was performed using X-ray diffraction and Raman spectroscopy. The bioactive glass was grinded in an agate mortar, and the powder was analyzed in a 2θ range between 10° and 60° using an Aeris-Panalytical diffractometer. The diffractometer worked at 40 kV and 14 mA, and CuKα radiation (λ = 1.54056 Å) was used.

Raman spectroscopy was conducted on the bulk samples using a Jobin Yvon HR800 spectrometer, Kyoto, Japan, equipped with an Ar^+^ laser (λ = 532 nm). The spectra were acquired in back-scattering geometry, spanning the spectral range from 200 to 1400 cm^−1^. The deconvolution process was performed using the OriginLab 2021 software.

Morphology characterization was performed on the bulk surface using a Vega 3 TESCAN SEM microscope, Brno, Czech Republic.

### 2.3. Electrical Characterization 

Electrical measurements were performed on glass bulk samples with a uniform thickness of 1 mm. The surface area of the samples was measured utilizing ImageJsoftware 1.8.0 [51]. Subsequently, the opposing parallel surfaces of the samples were painted with a layer of silver conducting paste. Both direct current (dc) and alternating current (ac) analyses were conducted in a nitrogen bath cryostat, which allows for measurements in the temperature range from 100 to 400 K to be obtained. An Oxford Research IT-C4 system with temperature monitoring enabled by a platinum sensor was used to control the sample temperature. The dc conductivity was measured with a Keithley electrometer model 617A, applying a voltage of 100 V across the bulk glass. In the case of ac experiments, dielectric spectroscopy measurements were performed using an impedance analyzer, Agilent 4294A, Santa Clara, CA, United States. The measurements were carried out within a broad frequency range between 100 Hz and 1 MHz, employing the C_p_−R_p_ configuration and applying an ac signal of 0.5 V. The complex electric permittivity ε* and the complex dielectric modulus M* formalisms were determined using Equation (1) and Equation (2) [54,55,56]:

ε* = ε′ − j ε″ = C_p_ (d/ε_0_ A) − j d (ω R_p_ ε_0_ A), (1)

M* = 1/ε* = M′+ iM″ = ε′/(ε′^2^+ ε″^2^) + I ε″/(ε′^2^+ ε″^2^), (2)

where C_p_ and R_p_ are the measured capacitance and resistance, d is the sample’s thickness, A is the electrode area, ε_0_ is the vacuum permittivity (8.8542 × 10^−12^ F/m), and ω is the angular frequency.

AC conductivity was calculated using the relation [51,56]:

σ_ac_* = ε_0_ ω ε″+ j ε_0_ ω ε′, (3)

The activation energy was determined by adjusting the temperature dependence of electrical conductivity using the Arrhenius model [54,56,57]:

σ = σ_0_ exp (−E_A_/(k_B_ T)), (4)

where σ_0_ is a pre-exponential factor, E_A_ is the activation energy, K_B_ is the Boltzmann constant, and T is the temperature. The activation energy, E_A_, is therefore determined from the slope of the graph ln (σ) versus 1/T.

## 3. Results and Discussion

**Figure 1:**
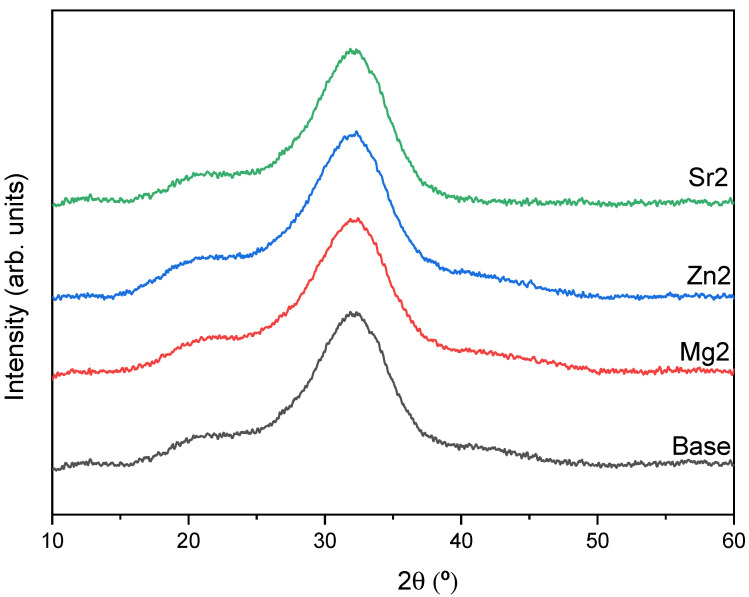
X-ray diffraction patterns of the bioglasses (base, Mg2, Zn2, and Sr2).

Figure 1 displays the XRD diffractograms for the base and the bioglasses with the highest concentration of MgO, ZnO, and SrO. The results show the typical amorphous behavior of 45S5 Bioglass. According to the literature, insertion of the oxides studied up to 2 mol% does not induce the formation of crystalline phases [58,59,60,61,62].

**Figure 2:**
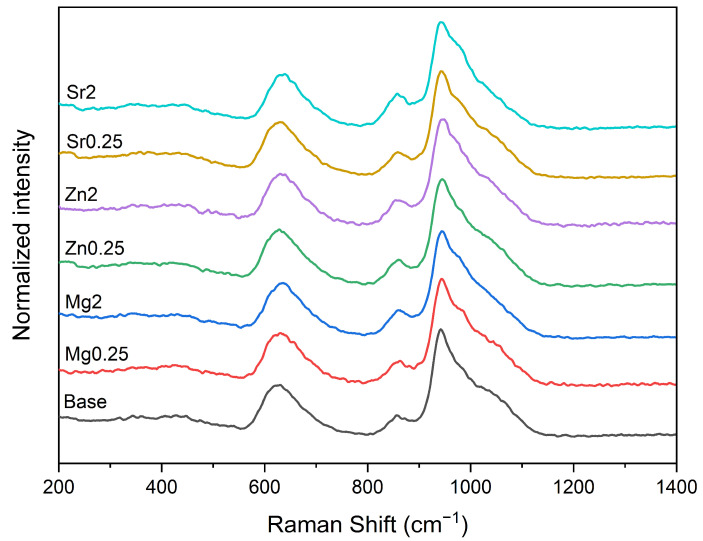
Raman spectra of the bioglass samples.

The Raman spectra of the prepared bioglasses are depicted in Figure 2, demonstrating a consistent trend among these samples. For silicate glasses, the most intriguing vibrational modes are those associated with symmetric and asymmetric stretching in the high-frequency region (800 and 1200 cm^−1^), regarded as especially significant. 

**Figure 3:**
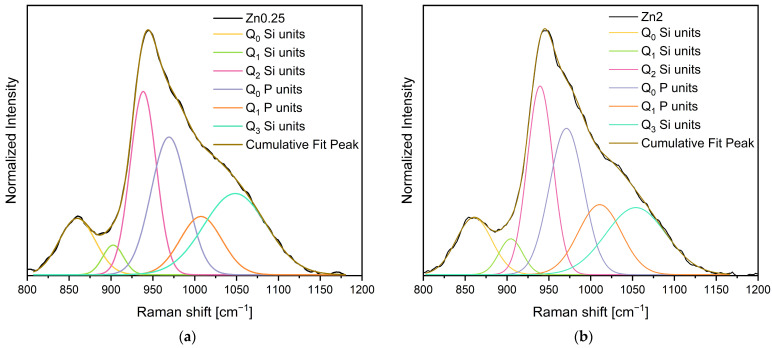
Deconvolution of Raman spectra for (**a**) Zn0.25 and (**b**) Zn2 bioglasses.

To conduct a more comprehensive analysis, Gaussian fitting was applied to deconvolve the Raman spectra in this region. Figure 3 illustrates the deconvolution of the Raman spectra for bioglasses containing 0.25 and 2% mol of ZnO. Six distinguishable vibrational modes can be found at 860–865 cm^−1^, 903–910 cm^−1^, 938–942 cm^−1^, 965–973 cm^−1^, 1001–1010 cm^−1^, and 1050–1065 cm^−1^, which correspond to Q_0_ Si units, Q_1_ Si units, Q_2_ Si, Q_0_ P and Q_1_ P units, and Q_3_ Si units, respectively [63,64,65,66,67]. The areas of the bands obtained using the deconvolution of Raman spectra for the Q_n_ units detected are represented in Table 1. The results obtained in Table 1 reveal the effect of oxides insertion.

**Figure 4:**
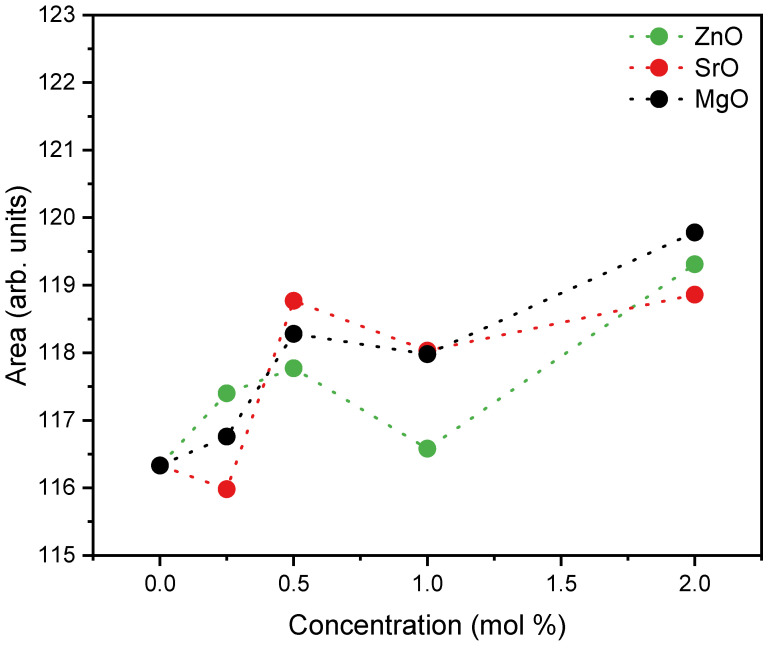
Sum of the areas of the Raman vibration bands associated with NBOs, Q_0_+ Q_1_ + Q_2_ + Q_3_, of Si and P units.

Figure 4 shows the sum of the area of Raman vibration bands associated with non-bridging oxygen (NBOs), i.e., Q_0_, Q_1_, Q_2_, and Q_3_ of the Si and P units for all samples. The results demonstrate that the insertion of ZnO, MgO, and SrO oxides into the bioglass network does not change significantly the amount of NBOs compared to the base sample. However, an increase in the concentration of these oxides from 1.0 to 2.0% suggests an increasing tendency of NBOs.

**Figure 5:**
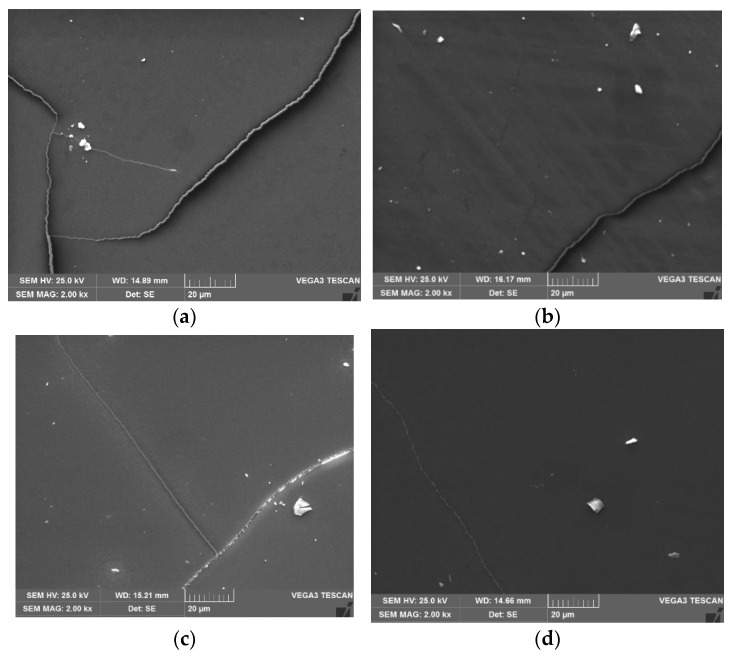
SEM micrographs for (**a**) base; (**b**) Zn2; (**c**) Sr2; and (**d**) Mg2 (magnification: 2 kx).

Figure 5 shows the micrographs of the bioglasses surface of the base and the bioglass with 2 mol% of ZnO, SrO, and MgO. The morphology confirms its glassy structure. The imperfections present on the surface of the bulk were created by the production method and demonstrated to be useful for acquiring the image in SEM.

**Figure 6:**
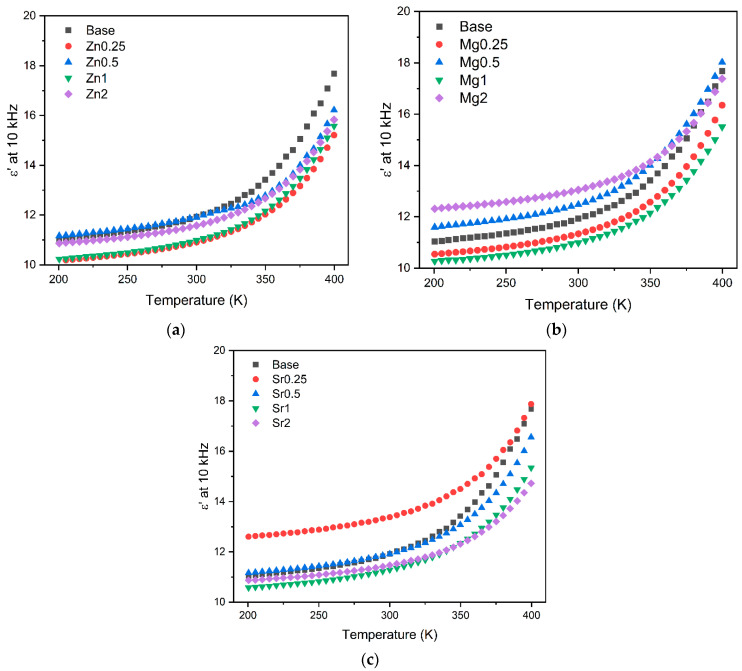
The dielectric constant, ε′, as a function of the temperature at 10 kHz of the glasses modified by the addition of (**a**) ZnO; (**b**) MgO; and (**c**) SrO.

Figure 6 depicts the temperature dependence of the dielectric constant, ε′, for all samples at a frequency of 10 kHz. The results show an increase in the dielectric constant with increasing temperature, which is likely attributed to the enhanced mobility of the dipoles from the glass matrix [19,47]. Based on the analysis of Figure 4 and Table 2, it is evident that the introduction of oxides did not yield a significant alteration in the dielectric constant when compared to the bioglass base.

**Figure 7:**
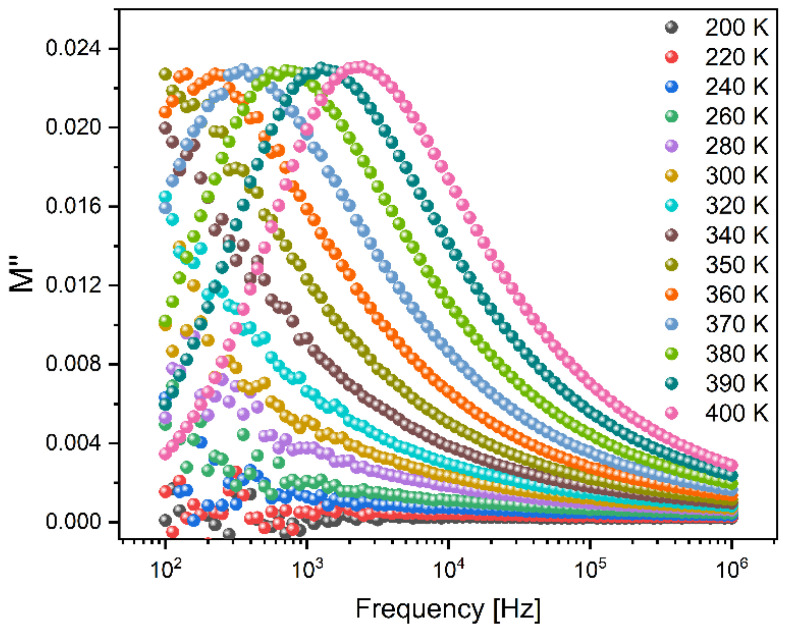
The imaginary part of the electric modulus, M″, versus the frequency for the Mg2 bioglass measured between 200 K and 400 K.

The dielectric properties of all the samples were analyzed using the modulus formalism, denoted as M*, defined as 1/ε*. This approach offers the advantage of minimizing the impact of low capacitance contributions, such as electrode polarization and low-frequency conductivity [19]. Figure 7 shows the variation in the imaginary part of the electric modulus versus the frequency at different temperatures for the Mg2 sample. Similar behavior was observed for all the prepared bioglass samples, indicating the presence of one dielectric relaxation process that shifts toward higher frequencies with increasing temperature. This particular dielectric relaxation behavior was not revealed by other approaches like permittivity, impedance, or admittance. Consequently, it can be deduced that the observed relaxation phenomenon is associated with an intrinsic characteristic related to the formation of dipoles between network modifiers and structurally proximate non-bridging oxygens.

**Figure 8:**
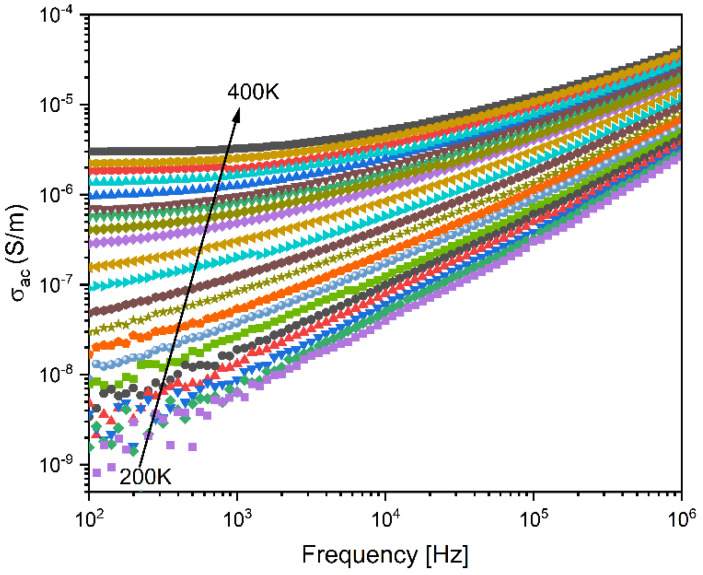
Frequency dependence of the ac conductivity at various temperatures for 45S5 bioglass measured in the temperature range between 200 and 400 K.

Conductivity and the dynamics of charge carriers can be better understood by studying the frequency dependence of ac conductivity (σ_ac_). Figure 8 shows the variation in σ_ac_ versus frequency for 45S5 bioglass.

At high temperatures, the variation in ac consists of two distinct regions. The initial one, situated in the region of low frequencies, is typically frequency-independent (horizontal plateau nature), giving rise to dc conductivity σ_dc_ resulting from the random diffusion of the ionic charge carriers through an activated hopping process. However, at a high frequency range, the conductivity increases linearly following a power law Aω^s^. Therefore, Jonscher’s model could be applied to describe the variation in the ac conductivity [68]:

σ_ac_ = σ_dc_ + Aω^s^, (5)

where σ_dc_ is the direct current conductivity, ω is the angular frequency, and A is a constant which determines the strength of polarizability. The exponent s in the equation represents the degree of interaction between mobile ions and their surrounding lattices. According to Funke et al. [69], the value of s holds physical significance. If s < 1, it implies that the load carriers undergo translational movement with a sudden jump. Conversely, when s > 1, it signifies a localized jump of the species (small jump without leaving the neighborhood).

**Figure 9:**
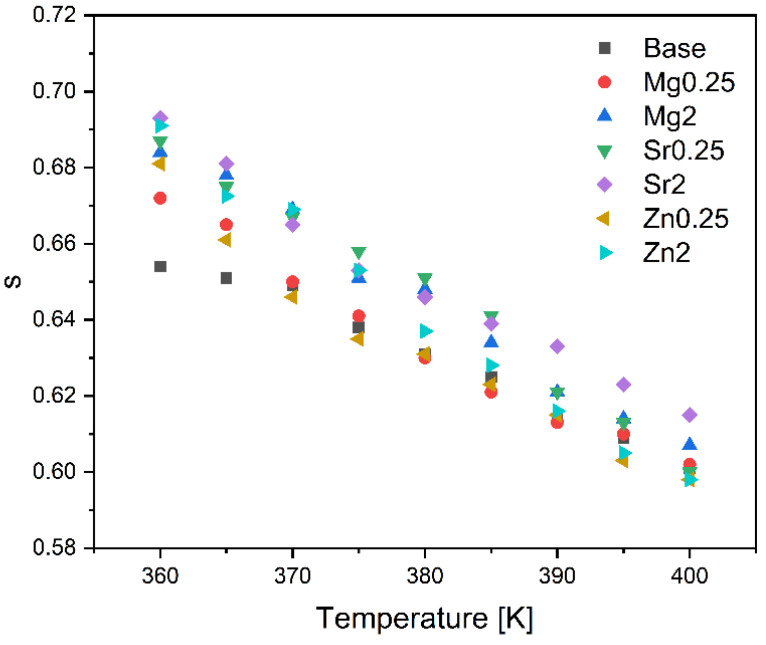
Temperature dependence of the frequency exponent s.

To determine the conduction mechanism of ac conductivity for the prepared bioglass samples, the variation in the s exponent as a function of the temperature is reported in Figure 9. Various models have been considered to describe the variation in the s exponent with temperature. These models are based on two distinct processes, specifically, quantum–mechanical tunneling or classical hopping over a barrier, or a variant or combination of the two [70]. Carriers have been assumed to be atoms or electrons (or polarons). As can be seen in Figure 9, the exponent s decreases with temperature for all the samples, proving that the ac conduction mechanism refers to correlated barrier hopping (CBH) [71,72]. According to this model, the charge carrier leaps between the sites over the potential barrier separating them.

**Figure 10:**
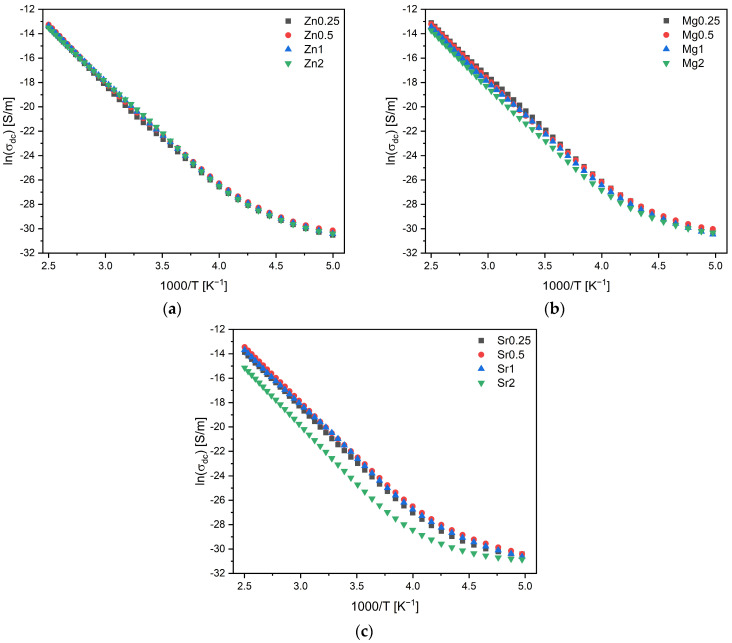
The logarithm of dc conductivity versus 1000/T of bioglasses modified by the insertion of (**a**) ZnO; (**b**) MgO; and (**c**) SrO.

Figure 10 illustrates the variation in the dc conductivity versus 1000/T on a logarithmic scale. It must be mentioned that the results below 100 K are not showed because of the low signal–noise ratio. It is clear that conductivity increases with temperature due to the enhanced mobility of charge carriers. Beyond approximately 270 K, this variation becomes linear, suggesting that the activation energy associated with this thermally activated process can be calculated using the Arrhenius formalism. At these temperature ranges, the ionic contribution predominates over the electronic contribution in glasses. Therefore, the conductivity of the bioglasses is associated with the energy carried by the network modifier (NaO, CaO, and ZnO, or MgO or SrO) moving through the glass network [45,73].

**Figure 11:**
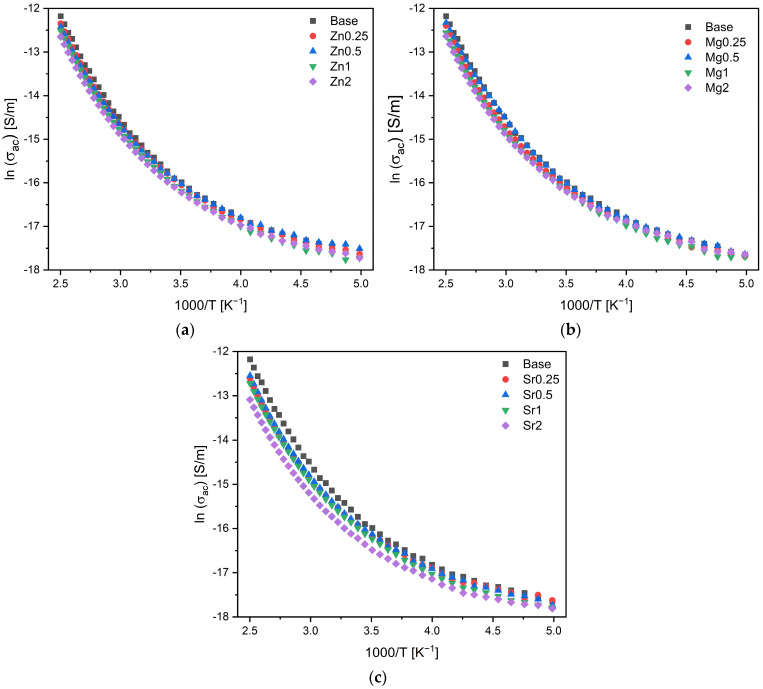
The logarithm of ac conductivity versus 1000/T at 10 kHz of bioglasses modified by the insertion of (**a**) ZnO; (**b**) MgO; and (**c**) SrO.

The temperature dependence of ac conductivity, as illustrated in Figure 11, exhibited a similar feature to that of dc conductivity. This suggests that the responsible units are similar to those in dc conductivity. The calculated activation energy for both ac and dc conductivity is reported in Table 2. The results show that the activation energy for dc conductivity is higher than that of ac conductivity. Furthermore, there are no discernible differences in activation energy across the samples. Consistent results are obtained for permittivity, which shows that the insertion of oxides does not induce a significant alteration. This finding demonstrates that the electrical charge storage property of 45S5 bioglass is not compromised by the addition of ZnO, MgO, or SrO at a concentration up to 2% mol.

## 4. Conclusions

A series of 45S5 bioactive glass modified by the addition of ZnO, MgO, and SrO was successfully produced using the melt-quenching technique. The Raman results did not show new vibration modes with the insertion of oxides until 2% mol. Nevertheless, the deconvolution of Raman spectra revealed that the incorporation of ZnO, MgO, and SrO into the bioglass network increases the amount of NBOs. However, increasing these oxides does not significantly impact NBOs levels. The electric response of the prepared glasses in the ac and dc regime did not show substantial changes with the addition of oxides. This is related to the insignificant change in the amount of NBO detected by Raman, probably associated with the low quantity of the added ZnO, MgO, or SrO. These results suggest that the potential of the bioglass to store electrical charge will not be compromised with the addition of ZnO, MgO, or SrO up to 2% mol. Therefore, all the prepared glasses have the ability to function as electrically charged coatings, one of the parameters that can induce a positive response in the bioactivity rate and consequently promote the bone regeneration process.

## Figures and Tables

**Table 1:** The areas of the bands for all Q_n_ units obtained by Raman spectra fitting.

Sample	Q_0_ Si Units	Q_1_ Si Units	Q_2_ Si Units	Q_0_ P Units	Q_1_ P Units	Q_3_ Si Units
Base	7.8	2.13	15.9	49.9	3.9	36.7
Zn0.25	12.29	3.71	28.81	29.82	14.65	28.25
Zn0.5	13.23	3.66	30.47	26.53	14.97	28.91
Zn1	13.53	4.48	29.29	26.04	11.55	31.69
Zn2	12.31	5.08	29.18	30.18	18.16	24.40
Mg0.25	12.24	3.12	32.67	26.34	14.36	27.25
Mg0.5	13.45	3.15	33.96	24.75	14.40	29.06
Mg1	13.34	3.44	32.10	25.08	16.26	27.81
Mg2	14.20	5.09	28.91	27.11	16.77	26.78
Sr0.25	13.14	3.17	29.53	30.09	15.52	25.31
Sr0.5	14.58	4.23	24.41	31.41	18.12	25.53
Sr1	15.26	4.47	27.98	31.77	17.31	21.19
Sr2	17.98	3.93	32.41	29.42	17.22	18.82

**Table 2:** The dc conductivity (σ_dc_), dc activation energy, ac conductivity (σ_ac_), ac activation energy, dielectric constant (ε′), and dielectric loss (tan δ) at 10 kHz and 300 K.

Sample	σ_dc_ (×10^−10^) [S/m]	E_a dc_ [kJ/mol]	σ_ac_ (×10^−7^) [S/m]	E_a ac_ [kJ/mol]	ε′	tan δ
Base	6.9 ± 0.10	77.7 ± 0.80	1.73 ± 0.02	40.99 ± 0.43	11.9 ± 0.7	0.026 ± 0.003
Zn0.25	5.7 ± 0.13	80.5 ± 0.07	1.27 ± 0.02	42.27 ± 1.70	11.0 ± 0.7	0.024 ± 0.003
Zn0.5	8.1 ± 0.16	79.0 ± 0.13	1.30 ± 0.02	40.42 ± 1.03	11.9 ± 0.7	0.019 ± 0.002
Zn1	8.7 ± 0.22	75.8 ± 0.15	1.41 ± 0.03	39.80 ± 0.71	11.0 ± 0.8	0.023 ± 0.004
Zn2	10.5 ± 0.26	73.5 ± 0.29	1.31 ± 0.02	38.75 ± 0.76	11.6 ± 0.9	0.020 ± 0.003
Mg0.25	10.4 ± 0.51	72.8 ± 0.75	1.74 ± 0.04	40.67 ± 0.42	11.3 ± 1.0	0.023 ± 0.004
Mg0.5	10.1 ± 0.22	75.3 ± 0.78	1.68 ± 0.03	37.65 ± 0.39	12.5 ± 0.8	0.024 ± 0.003
Mg1	10.2 ± 0.31	75.5 ± 0.78	1.36 ± 0.03	39.54 ± 0.41	11.0 ± 0.8	0.022 ± 0.003
Mg2	5.7 ± 0.13	78.1 ± 0.81	1.33 ± 0.03	39.11 ± 0.40	13.1 ± 0.8	0.018 ± 0.003
Sr0.25	4.9 ± 0.12	76.2 ± 0.79	1.34 ± 0.02	39.00 ± 0.40	13.4 ± 0.4	0.018 ± 0.002
Sr0.5	7.6 ± 0.23	75.5 ± 0.78	1.42 ± 0.02	39.45 ± 0.41	12.1 ± 0.1	0.021± 0.003
Sr1	7.8 ± 0.21	74.6 ± 0.77	1.30 ± 0.02	37.93 ± 0.40	11.3 ± 0.2	0.021 ± 0.002
Sr2	0.9 ± 0.02	79.7 ± 0.83	1.03 ± 0.01	39.94 ± 0.40	11.8 ± 0.7	0.015 ± 0.002

## Data Availability

The data presented in this study are available from the corresponding author upon request.

## References

[1] Petersen R.C. (2014). Titanium Implant Osseointegration Problems with Alternate Solutions Using Epoxy/Carbon-Fiber-Reinforced Composite. Metals.

[2] Silva R.C.S., Agrelli A., Andrade A.N., Mendes-Marques C.L., Arruda I.R.S., Santos L.R.L., Vasconcelos N.F., Machado G. (2022). Titanium Dental Implants: An Overview of Applied Nanobiotechnology to Improve Biocompatibility and Prevent Infections. Materials.

[3] Kearns V.R., Williams R.L., Mirvakily F., Doherty P.J., Martin N. (2013). Guided Gingival Fibroblast Attachment to Titanium Surfaces: An in Vitro Study. J. Clin. Periodontol..

[4] Wang Q., Zhou P., Liu S., Attarilar S., Ma R.L.-W., Zhong Y., Wang L. (2020). Multi-Scale Surface Treatments of Titanium Implants for Rapid Osseointegration: A Review. Nanomaterials.

[5] Guglielmotti M.B., Olmedo D.G., Cabrini R.L. (2019). Research on Implants and Osseointegration. Periodontology 2000.

[6] Pádua A.S., Gavinho S.R., Vieira T., Hammami I., Silva J.C., Borges J.P., Graça M.P.F. (2023). In Vitro Characterization of Doped Bioglass 45S5/HAp Coatings Obtained by CoBlastTM Deposition. Coatings.

[7] Lung C.Y.K., Abdalla M.M., Chu C.H., Yin I., Got S.-R., Matinlinna J.P. (2021). A Multi-Element-Doped Porous Bioactive Glass Coating for Implant Applications. Materials.

[8] Davidson D.J., Spratt D., Liddle A.D. (2019). Implant Materials and Prosthetic Joint Infection: The Battle with the Biofilm. EFORT Open Rev..

[9] Priyadarshini B., Rama M., Chetan, Vijayalakshmi U. (2019). Bioactive Coating as a Surface Modification Technique for Biocompatible Metallic Implants: A Review. J. Asian Ceram. Soc..

[10] Zafar M.S., Farooq I., Awais M., Najeeb S., Khurshid Z., Zohaib S., Kaur G. (2019). Chapter 11-Bioactive Surface Coatings for Enhancing Osseointegration of Dental Implants. Biomedical, Therapeutic and Clinical Applications of Bioactive Glasses.

[11] Aswad M.A., Sabree I.K., Abd A.H.S. (2021). An Examination of the Effect of Adding Zirconia to Bioactive Glass-Ceramic Properties. IOP Conf. Ser. Mater. Sci. Eng..

[12] Joy-anne N.O., Su Y., Lu X., Kuo P.-H., Du J., Zhu D. (2019). Bioactive Glass Coatings on Metallic Implants for Biomedical Applications. Bioact. Mater..

[13] Hench L.L. (2006). The Story of Bioglass^®^. J. Mater. Sci. Mater. Med..

[14] Hench L.L. (2013). An Introduction to Bioceramics.

[15] Hench L.L., Greenspan D. (2013). Interactions between Bioactive Glass and Collagen: A Review and New Perspectives. J. Aust. Ceram. Soc..

[16] Xynos I.D., Hukkanen M.V.J., Batten J.J., Buttery L.D., Hench L.L., Polak J.M. (2000). Bioglass ^®^45S5 Stimulates Osteoblast Turnover and Enhances Bone Formation In Vitro: Implications and Applications for Bone Tissue Engineering. Calcif. Tissue Int..

[17] Bano S., Romero A.R., Grant D.M., Nommeots-Nomm A., Scotchford C., Ahmed I., Hussain T. (2021). In-Vitro Cell Interaction and Apatite Forming Ability in Simulated Body Fluid of ICIE16 and 13–93 Bioactive Glass Coatings Deposited by an Emerging Suspension High Velocity Oxy Fuel (SHVOF) Thermal Spray. Surf. Coat. Technol..

[18] Al Mugeiren O.M., Baseer M.A. (2019). Dental Implant Bioactive Surface Modifiers: An Update. J. Int. Soc. Prev. Community Dent..

[19] Hammami I., Gavinho S.R., Pádua A.S., Sá-Nogueira I., Silva J.C., Borges J.P., Valente M.A., Graça M.P.F. (2023). Bioactive Glass Modified with Zirconium Incorporation for Dental Implant Applications: Fabrication, Structural, Electrical, and Biological Analysis. Int. J. Mol. Sci..

[20] Joy-anne N.O., Akande O., Ecker M. (2021). Incorporation of Novel Elements in Bioactive Glass Compositions to Enhance Implant Performance. Current Concepts in Dental Implantology-From Science to Clinical Research.

[21] Xynos I.D., Edgar A.J., Buttery L.D., Hench L.L., Polak J.M. (2000). Ionic Products of Bioactive Glass Dissolution Increase Proliferation of Human Osteoblasts and Induce Insulin-like Growth Factor II mRNA Expression and Protein Synthesis. Biochem. Biophys. Res. Commun..

[22] Silver I.A., Deas J., Erecińska M. (2001). Interactions of Bioactive Glasses with Osteoblasts in Vitro: Effects of 45S5 Bioglass^®^, and 58S and 77S Bioactive Glasses on Metabolism, Intracellular Ion Concentrations and Cell Viability. Biomaterials.

[23] Kang T.-Y., Seo J.-Y., Ryu J.-H., Kim K.-M., Kwon J.-S. (2021). Improvement of the Mechanical and Biological Properties of Bioactive Glasses by the Addition of Zirconium Oxide (ZrO_2_) as a Synthetic Bone Graft Substitute. J. Biomed. Mater. Res. Part A.

[24] Hammami I., Gavinho S.R., Pádua A.S., Graça M.P.F., Silva J.C. Synthesis and Characterization of Iron Containing Bioactive Glass for Implants. Proceedings of the 2022 E-Health and Bioengineering Conference (EHB).

[25] Gavinho S.R., Pádua A.S., Sá-Nogueira I., Silva J.C., Borges J.P., Costa L.C., Graça M.P.F. (2022). Biocompatibility, Bioactivity, and Antibacterial Behaviour of Cerium-Containing Bioglass^®^. Nanomaterials.

[26] Balamurugan A., Balossier G., Laurent-Maquin D., Pina S., Rebelo A.H.S., Faure J., Ferreira J.M.F. (2008). An in Vitro Biological and Anti-Bacterial Study on a Sol-Gel Derived Silver-Incorporated Bioglass System. Dent. Mater..

[27] Akhtach S., Tabia Z., El Mabrouk K., Bricha M., Belkhou R. (2021). A Comprehensive Study on Copper Incorporated Bio-Glass Matrix for Its Potential Antimicrobial Applications. Ceram. Int..

[28] Hammami I., Gavinho S.R., Jakka S.K., Valente M.A., Graça M.P.F., Pádua A.S., Silva J.C., Sá-Nogueira I., Borges J.P. (2023). Antibacterial Biomaterial Based on Bioglass Modified with Copper for Implants Coating. J. Funct. Biomater..

[29] Bargavi P., Chitra S., Durgalakshmi D., Radha G., Balakumar S. (2020). Zirconia Reinforced Bio-Active Glass Coating by Spray Pyrolysis: Structure, Surface Topography, in-Vitro Biological Evaluation and Antibacterial Activities. Mater. Today Commun..

[30] Cacciotti I. (2017). Bivalent Cationic Ions Doped Bioactive Glasses: The Influence of Magnesium, Zinc, Strontium and Copper on the Physical and Biological Properties. J. Mater. Sci..

[31] Palanikumar L., Ramasamy S.N., Balachandran C. (2014). Size-Dependent Antimicrobial Response of Zinc Oxide Nanoparticles. IET Nanobiotechnology.

[32] Ansari M.A., Khan H.M., Khan A.A., Sultan A., Azam A. (2012). Characterization of Clinical Strains of MSSA, MRSA and MRSE Isolated from Skin and Soft Tissue Infections and the Antibacterial Activity of ZnO Nanoparticles. World J. Microbiol. Biotechnol..

[33] Malka E., Perelshtein I., Lipovsky A., Shalom Y., Naparstek L., Perkas N., Patick T., Lubart R., Nitzan Y., Banin E. (2013). Eradication of Multi-Drug Resistant Bacteria by a Novel Zn-Doped CuO Nanocomposite. Small.

[34] Gavinho S.R., Pádua A.S., Sá-Nogueira I., Silva J.C., Borges J.P., Costa L.C., Graça M.P.F. (2023). Fabrication, Structural and Biological Characterization of Zinc-Containing Bioactive Glasses and Their Use in Membranes for Guided Bone Regeneration. Materials.

[35] Tabia Z., Mabrouk K.E., Bricha M., Nouneh K. (2019). Mesoporous Bioactive Glass Nanoparticles Doped with Magnesium: Drug Delivery and Acellular in Vitro Bioactivity. RSC Adv..

[36] Moghanian A., Sedghi A., Ghorbanoghli A., Salari E. (2018). The Effect of Magnesium Content on in Vitro Bioactivity, Biological Behavior and Antibacterial Activity of Sol–Gel Derived 58S Bioactive Glass. Ceram. Int..

[37] Coelho C.C., Padrão T., Costa L., Pinto M.T., Costa P.C., Domingues V.F., Quadros P.A., Monteiro F.J., Sousa S.R. (2020). The Antibacterial and Angiogenic Effect of Magnesium Oxide in a Hydroxyapatite Bone Substitute. Sci. Rep..

[38] Damian-Buda A.I., Voicu G., Vasile B.S., Banciu A., Iordache F., Ciocan L.T. (2022). Development of Mesoporous Borosilicate Bioactive Glass Nanoparticles Containing Mg^2+^ and Zn^2+^: Biocompatibility, Bioactivity and Antibacterial Activity. J. Non-Cryst. Solids.

[39] Gavinho S.R., Pádua A.S., Holz L.I.V., Sá-Nogueira I., Silva J.C., Borges J.P., Valente M.A., Graça M.P.F. (2023). Bioactive Glasses Containing Strontium or Magnesium Ions to Enhance the Biological Response in Bone Regeneration. Nanomaterials.

[40] Mosaddad S.A., Yazdanian M., Tebyanian H., Tahmasebi E., Yazdanian A., Seifalian A., Tavakolizadeh M. (2020). Fabrication and Properties of Developed Collagen/Strontium-Doped Bioglass Scaffolds for Bone Tissue Engineering. J. Mater. Res. Technol..

[41] Chaichana W., Insee K., Chanachai S., Benjakul S., Aupaphong V., Naruphontjirakul P., Panpisut P. (2022). Physical/Mechanical and Antibacterial Properties of Orthodontic Adhesives Containing Sr-Bioactive Glass Nanoparticles, Calcium Phosphate, and Andrographolide. Sci. Rep..

[42] Manoochehri H., Ghorbani M., Moosazadeh Moghaddam M., Nourani M.R., Makvandi P., Sharifi E. (2022). Strontium Doped Bioglass Incorporated Hydrogel-Based Scaffold for Amplified Bone Tissue Regeneration. Sci. Rep..

[43] Mutreja I., Kumar D., Hogan K., Campbell E., Mansky K., Aparicio C. (2022). Strontium- and Peptide-Modified Silicate Nanostructures for Dual Osteogenic and Antimicrobial Activity. Biomater. Adv..

[44] Kumar A., Banrjee S., Roy P., Xu H., Mariappan C.R. (2022). Osteogenic Commitment of Strontium Nanoparticles Doped Mesoporous Bioactive Glass-Ceramics. Mater. Sci. Eng. B.

[45] Obata A., Nakamura S., Moriyoshi Y., Yamashita K. (2003). Electrical Polarization of Bioactive Glass and Assessment of Their in Vitro Apatite Deposition. J. Biomed. Mater. Res. Part A.

[46] Dubey A.K., Oyama Y., Kakimoto K. (2019). Surface Charge-Assisted Synthesis of ZnO on Polarized BaTiO3 Substrate. Ionics.

[47] Obata A., Nakamura S., Yamashita K. (2004). Interpretation of Electrical Polarization and Depolarization Mechanisms of Bioactive Glasses in Relation to Ionic Migration. Biomaterials.

[48] Obata A., Nakamura S., Sekijima Y., Yamashita K. (2004). Control of Surface Reactions of Bioactive Glass by Electrical Polarization. J. Ceram. Soc. Jpn..

[49] Kobayashi M., Saito H., Mase T., Sasaki T., Wang W., Tanaka Y., Nakamura M., Nagai A., Yamashita K. (2010). Polarization of Hybridized Calcium Phosphoaluminosilicates with 45S5-Type Bioglasses. Biomed. Mater..

[50] Guo W., Liu Y., Zhu X., Wang S. (2011). Temperature-Dependent Dielectric Properties of Honey Associated with Dielectric Heating. J. Food Eng..

[51] Gavinho S.R., Melo B.M.G., Borges J.P., Silva J.C., Graça M.P.F. (2023). Thermal, Structural, Morphological and Electrical Characterization of Cerium-Containing 45S5 for Metal Implant Coatings. Coatings.

[52] Yamashita K., Oikawa N., Umegaki T. (1996). Acceleration and Deceleration of Bone-Like Crystal Growth on Ceramic Hydroxyapatite by Electric Poling. Chem. Mater..

[53] Verma A.S., Kumar D., Dubey A.K. (2020). Antibacterial and Cellular Response of Piezoelectric Na0.5K0.5NbO3modified 1393 Bioactive Glass. Mater. Sci. Eng. C.

[54] Graça M.P.F., Ferreira Da Silva M.G., Valente M.A. (2007). Preparation, Structure, Morphology, and Dc and Ac Conductivity of the 88SiO2-6Li2O-6Nb2O5 (% Mole) Sol-Gel Derived Glass-Ceramics. J. Sol-Gel Sci. Technol..

[55] El-Mallawany R.A. (2014). Tellurite Glasses Handbook: Physical Properties and Data.

[56] Barsoukov E., Macdonald J.R. (2018). Impedance Spectroscopy: Theory, Experiment, and Applications.

[57] Macdonald J.R. (1987). Emphasizing Solid Materials and Systems. Impedance Spectroscopy.

[58] Miola M., Verné E., Ciraldo F.E., Cordero-Arias L., Boccaccini A.R. (2015). Electrophoretic Deposition of Chitosan/45S5 Bioactive Glass Composite Coatings Doped with Zn and Sr. Front. Bioeng. Biotechnol..

[59] Sergi R., Bellucci D., Salvatori R., Maisetta G., Batoni G., Cannillo V. (2019). Zinc Containing Bioactive Glasses with Ultra-High Crystallization Temperature, Good Biological Performance and Antibacterial Effects. Mater. Sci. Eng. C.

[60] Neščáková Z., Zheng K., Liverani L., Nawaz Q., Galusková D., Kaňková H., Michálek M., Galusek D., Boccaccini A.R. (2019). Multifunctional Zinc Ion Doped Sol-Gel Derived Mesoporous Bioactive Glass Nanoparticles for Biomedical Applications. Bioact. Mater..

[61] Taherkhani S., Moztarzadeh F. (2016). Influence of Strontium on the Structure and Biological Properties of Sol–Gel-Derived Mesoporous Bioactive Glass (MBG) Powder. J. Sol-Gel Sci. Technol..

[62] Zhao R., Shi L., Gu L., Qin X., Song Z., Fan X., Zhao P., Li C., Zheng H., Li Z. (2021). Evaluation of Bioactive Glass Scaffolds Incorporating SrO or ZnO for Bone Repair: In Vitro Bioactivity and Antibacterial Activity. J. Appl. Biomater. Funct. Mater..

[63] Dziadek M., Zagrajczuk B., Jelen P., Olejniczak Z., Cholewa-Kowalska K. (2016). Structural Variations of Bioactive Glasses Obtained by Different Synthesis Routes. Ceram. Int..

[64] Araujo M.S., Silva A.C., Bartolomé J.F., Mello-Castanho S. (2020). Structural and Thermal Behavior of 45S5 Bioglass^®^-Based Compositions Containing Alumina and Strontium. J. Am. Ceram. Soc..

[65] Aguiar H., Serra J., González P., León B. (2009). Structural Study of Sol-Gel Silicate Glasses by IR and Raman Spectroscopies. J. Non-Cryst. Solids.

[66] Berezicka A., Szumera M., Sułowska J., Jeleń P., Olejniczak Z., Stępień J., Zając M., Pollastri S., Olivi L. (2022). Unraveling the Nature of Sulfur-Bearing Silicate-Phosphate Glasses: Insights from Multi-Spectroscopic (Raman, MIR, 29Si, 31P MAS-NMR, XAS, XANES) Investigation. Ceram. Int..

[67] Sun Y., Zhang Z., Liu L., Wang X. (2015). FTIR, Raman and NMR Investigation of CaO–SiO_2_–P_2_O_5_ and CaO–SiO_2_–TiO_2_–P_2_O_5_ Glasses. J. Non-Cryst. Solids.

[68] Jonscher A.K., Réau J.-M. (1978). Analysis of the Complex Impedance Data for β-PbF 2. J. Mater. Sci..

[69] Funke K. (1993). Jump Relaxation in Solid Electrolytes. Prog. Solid State Chem..

[70] Bechir M.B., Karoui K., Tabellout M., Guidara K., Rhaiem A.B. (2014). Electric and Dielectric Studies of the [N (CH_3_) 3H] 2CuCl_4_ Compound at Low Temperature. J. Alloys Compd..

[71] Ghosh A. (1990). Frequency-Dependent Conductivity in Bismuth-Vanadate Glassy Semiconductors. Phys. Rev. B.

[72] Austin I.G., Mott N.F. (1969). Polarons in Crystalline and Non-Crystalline Materials. Adv. Phys..

[73] Keshri S.R., Ganisetti S., Kumar R., Gaddam A., Illath K., Ajithkumar T.G., Balaji S., Annapurna K., Nasani N., Krishnan N.M.A. (2021). Ionic Conductivity of Na_3_Al_2_P_3_O_12_ Glass Electrolytes—Role of Charge Compensators. Inorg. Chem..

